# Enabling Next-Generation Public Safety Operations with Mission-Critical Networks and Wearable Applications †

**DOI:** 10.3390/s21175790

**Published:** 2021-08-28

**Authors:** Salwa Saafi, Jiri Hosek, Aneta Kolackova

**Affiliations:** 1 Department of Telecommunications, Faculty of Electrical Engineering and Communication, Brno University of Technology, Technicka 12, 616 00 Brno, Czech Republic; saafi@feec.vutbr.cz (S.S.); xkolac15@vutbr.cz (A.K.); 2 Unit of Electrical Engineering, Faculty of Information Technology and Communication Sciences, Tampere University, 33720 Tampere, Finland

**Keywords:** public safety, cellular connectivity, wearable technology, IoLST, mission-critical services

## Abstract

Public safety agencies have been working on the modernization of their communication networks and the enhancement of their mission-critical capabilities with novel technologies and applications. As part of these efforts, migrating from traditional land mobile radio (LMR) systems toward cellular-enabled, next-generation, mission-critical networks is at the top of these agencies’ agendas. In this paper, we provide an overview of cellular technologies ratified by the 3rd Generation Partnership Project (3GPP) to enable next-generation public safety networks. On top of using wireless communication technologies, emergency first responders need to be equipped with advanced devices to develop situational awareness. Therefore, we introduce the concept of the Internet of Life-Saving Things (IoLST) and focus on the role of wearable devices—more precisely, cellular-enabled wearables, in creating new solutions for enhanced public safety operations. Finally, we conduct a performance evaluation of wearable-based, mission-critical applications. So far, most of the mission-critical service evaluations target latency performance without taking into account reliability requirements. In our evaluation, we examine the impact of device- and application-related parameters on the latency and the reliability performance. We also identify major future considerations for better support of the studied requirements in next-generation public safety networks.

## 1. Introduction

Wireless communication technologies and equipment are particularly important for police officers, firefighters, and emergency medical service (EMS) workers. These emergency first responders rely on communication capabilities to exchange critical information and develop situational awareness in very challenging environments [1]. Hence, public safety organizations have to select appropriate communication technologies and solutions to provide their personnel with the required capabilities. To offer delay-sensitive, reliable, and secure services, public safety networks utilize dedicated communication systems based on land mobile radio (LMR) technologies. These include terrestrial trunked radio (TETRA), TETRA for police (TETRAPOL), and Project 25 (P25) of the Association of Public Safety Communications Officials (APCO) [2].

The main services provided by LMR-based networks to public safety to users are narrowband voice-centric services, such as priority calls with push-to-talk (PTT) functionalities. However, public safety operations are expected to leap to the next levels of efficiency by utilizing broadband data communications [3]. Having mature, multivendor, and multiservice infrastructures, commercial cellular networks are considered to be an alternative for LMR systems [4]. Deployment cost optimization and public safety service expansion are the main reasons considered by The Critical Communications Association (TCCA) to select the Long-Term Evolution (LTE) technology as the basis for future public safety implementations [5]. With the same perspective, we advocate the use of cellular connectivity for public safety communications and propose complementing these capabilities with wearable solutions for mission-critical applications.

In our previous work published in [6], we provided a state-of-the-art overview of cellular connectivity for public safety communications and wearable technology in the Internet of Life-Saving Things (IoLST). We then conducted a performance evaluation of a mission-critical service (i.e., mission-critical PTT (MCPTT)) using cellular-enabled wearables, with a focus on latency as the performance indicator. In the present work, we build upon the state of the art and extend the performance evaluation of [6] as follows:

- Identifying the solutions introduced by the 3rd Generation Partnership Project (3GPP) for the support of public safety communications. We review the identified solutions as enablers of cellular-based public safety networks, also referred to as next-generation public safety networks [4]. As part of this technology review, we track the enhancements of the identified technologies through the different 3GPP releases.
- Expanding and better illustrating the examples of IoLST use cases where cellular-enabled wearables can be employed.
- Complementing the latency evaluation in wearable-based MCPTT private and group calls with reliability results. We discuss the obtained results and shed light on the need for latency and reliability trade-off mechanisms in MCPTT applications.
- Providing standardization-related insights and future considerations for better support of the requirements of wearable-based MCPTT services in next-generation public safety networks.

The rest of the paper is organized as follows. Relevant research works dealing with wireless communication technologies and solutions for public safety are discussed in Section 2. By reviewing these works, we identify the research gaps that we aim to address in this work. In Section 3, we review the cellular technologies ratified by 3GPP for the support of mission-critical applications in next-generation public safety networks. Section 4 is dedicated to the discussion of the role of wearable technology in enabling new IoLST services. In the sequel, we assess latency and reliability performance in wearable-based MCPTT applications using system-level simulations. The performance evaluation methodology, results discussion, and future considerations are provided in Section 5. Finally, the conclusion is drawn in Section 6 with the main findings of this work.

## 2. Related Work

Although the study of communication capabilities for public safety has been in the scope of several research works, the overall number of these papers in the literature does not reflect the significance of the topic. In [7], the authors surveyed regulatory, standardization, and research activities dealing with wireless communication technologies in the public safety domain, with a particular focus on LMR systems. However, the inability of the traditionally used LMR systems to support modern data applications makes the migration toward standards that support the requirements of broadband services evident [8]. Consequently, the authors in [9] performed a comparative analysis of legacy (i.e., LMR) and emerging (i.e., LTE) technologies for public safety communications. The spectrum allocation for public safety usage across all the frequency bands in the United States and the benefits of LTE-based over LMR-based public safety networks were among the main topics addressed in [9].

LTE-based public safety networks were also studied in [10,11]. These works discussed how 4th Generation (4G) cellular technology promises to provide an evolution path toward broadband capabilities for existing and new public safety networks. As the next step after 4G, the 5th Generation (5G) standard brings massive improvements and novel capabilities for multiple vertical sectors including the public safety industry [12]. In [13], the authors presented a 5G solution proposed by the FASTER project for safe and efficient emergency response [14]. This solution was based on 5G nonstandalone (NSA) architecture and ultra-reliable low-latency communications (URLLC). A 5G-based, cloud-enabled, small-cell architecture was also proposed in [15] as part of the 5G ESSENCE project [16]. Cellular technologies are the communication solutions adopted in [10,11,12,13,14,15,16]. However, neither of these works provided an updated summary of the cellular features ratified by 3GPP to enable the deployment of 4G- and 5G-based public safety networks.

Other public-safety-related research works focused on the equipment and devices that can be used to assist first responders in public safety operations. The main assistant systems employed in public safety networks and proposed in several works are unmanned aerial vehicles (UAVs) or drones. The authors in [17,18] showed that public safety communications can benefit from deploying UAVs for providing broadband connectivity and extending network coverage and capacity. The ability to cover unreachable locations was the motivation behind the use of UAVs in a public safety solution for real-time immersive remote monitoring in [19].

Although the authors in [19,20] introduced using wearable devices by first responders, the presented use cases were limited to conventional high-end augmented/virtual reality (AR/VR) and low-end Internet of Things (IoT) applications. While AR/VR head-mounted devices were employed in [19] for immersive remote presence, ref. [20] proposed the utilization of IoT sensors and cameras in a collaborative system for disaster management. Other categories of wearables with potentially more applications, such as watch-type devices, are not included among the studied use cases. Therefore, a closer look at the potential role of wearable technology in cellular-enabled public safety networks is not provided in these works. In the scope of this paper, we aim to address the above-identified research gaps in terms of systematizing cellular communication technologies and wearable solutions for next-generation public safety operations.

## 3. Toward Next-Generation Public Safety Networks

The migration from LMR systems to next-generation public safety networks requires the introduction of essential solutions and features to ensure mission-critical grade performance. Hence, 3GPP has performed additional standardization efforts for the support of public safety services over cellular networks starting from its Rel-12 [5].

### 3.1. Public-Safety-Targeted Technologies

In Table 1, we summarize the main cellular technologies that have been ratified by 3GPP as a result of the public-safety-targeted standardization efforts. Group call system enablers (GCSE) provide a collection of mechanisms that enable both unicast and multicast transmissions for group communications. Proximity services (ProSe) were also among the main standardization items ratified in 3GPP Rel-12. They present the architecture and the radio interface for device-to-device (D2D) communications in cellular networks [5]. The new D2D interface is known as sidelink and was introduced as part of the support of public safety ProSe in LTE networks. As proved by several research works dealing with this technology, D2D communications allow cellular networks to take advantage of three gains (i.e., proximity, hop, and reuse) and several network performance improvements (i.e., high data rates, low delays, high reliability, and low power consumption) [21].

Additionally, establishing direct links between devices in out-of-coverage scenarios helps to provide first responders with the needed communications, especially in dangerous situations [22]. To support such scenarios, two LTE sidelink transmission modes were ratified by 3GPP for public safety. The sidelink transmission mode defines the entity responsible for the sidelink radio resource allocation: (i) mode-1, also called scheduled mode, in which the sidelink resource scheduling is monitored by the base station; (ii) mode-2, known as autonomous mode, where user equipments (UEs) rely on sidelink preconfigurations stored in the devices [23]. While in-coverage UEs can operate in mode-1 or mode-2 as decided by the network, out-of-coverage UEs are restricted to using mode-2.

As depicted in Table 1, the isolated E-UTRAN operation for public safety (IOPS) was ratified in 3GPP Rel-13. The aim behind its introduction is to provide public safety users with network access in challenging situations. The latter include cases where the network is overloaded due to increased user demands and where the access network part loses its backhaul connection with the core network in disaster areas [24]. While only voice-based communications are exchanged in LMR networks, public safety LTE (PS-LTE) networks offer a set of mission-critical services. These were introduced by 3GPP among its public-safety-related standardization items. Based on the market demands, MCPTT was the first major step in the series of mission-critical services introduced in Rel-13. 3GPP then added certain enhancements to the MCPTT specification in Rel-14. It also enriched its repertoire of standardized public safety applications by introducing mission-critical data (MCData) and mission-critical video (MCVideo) [25]. These three applications are also called MCX services.

**Figure 1:**
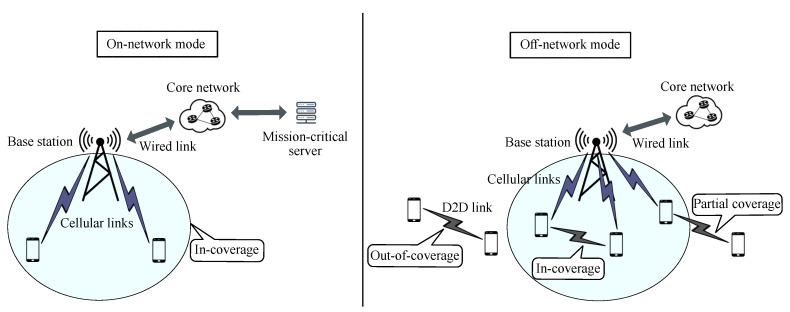
On-network and off-network functional modes for MCX services.

A general framework for MCX was provided in 3GPP Rel-14 to facilitate the standardization of additional services in the upcoming releases. This framework includes a common architecture for the support of MCX services. Two functional or architectural modes are defined based on the existence of a mission-critical server in the network: on-network and off-network modes [26]. Figure 1 shows the differences between the two modes. In on-network mode, the communications are based on a client/server setup. Client/server communications are established via the LTE core network. However, in off-network mode, the communications are only supported by UEs in a peer-to-peer setup [26]. The network coverage requirement in the two MCX architectures is also illustrated in Figure 1. To deploy the on-network mode, MCX users have to be within the coverage of the cellular network. On the contrary, off-network MCX users can establish D2D links in different network coverage situations (i.e., in-coverage, out-of-coverage, and partial coverage).

Although the standardization efforts on certain public safety technologies were frozen, 3GPP continues to introduce further enhancements for better support of public safety services over cellular networks. We outline, in Table 2, the main improvements beyond Rel-12 of public safety technologies. On top of the introduction of new discovery and direct communication features in the Evolved Packet System (EPS) [27], a major enhancement to ProSe is the support of UE-to-network relaying for IoT and wearables in 3GPP Rel-14 [28]. Supporting D2D connectivity in 5G networks, New Radio (NR) sidelink targets mainly Vehicle-to-Everything (V2X) services with solutions ratified in 3GPP Rel-16. However, Rel-17 NR sidelink enhancements for public safety include the support of ProSe communications and UE-to-network-relaying over 5G networks [29].

Another set of improvements of public-safety-targeted cellular solutions is related to MCX services. Table 2 summarizes the enhancements that are common to the three types of MCX applications (i.e., MCPTT, MCData, and MCVideo). They include mission-critical security-related functionalities in Rel-14 [30] and support of multimedia broadcast multicast services (MBMS) in Rel-15 [31]. Beyond Rel-16, improvements focus on enhancing the functional MCX architectures [32] and identifying further requirements of MCX services. Examples of further requirements are related to the support of multiple devices, location, security, and media quality [33]. Enhanced handling of MCPTT emergency alerts, support of simultaneous video sessions, and off-network file distribution are among the enhancements that target each type of MCX application separately [31].

In addition to the above-discussed solutions, next-generation public safety networks can make use of other cellular features that are not specifically ratified for public safety operations. Examples of these additional enabling technologies are examined in the following section.

### 3.2. Additional Enabling Technologies

**Figure 2:**
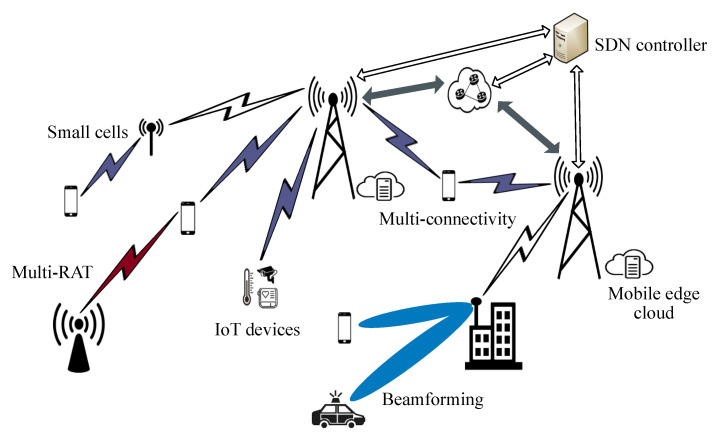
Examples of cellular technologies for next-generation public safety networks.

Figure 2 depicts examples of cellular technologies that can be deployed in next-generation public safety networks. Service reliability can be achieved not only using D2D communications but also through the flexible use of radio resources provided by multiconnectivity and multiradio access technologies (multi-RAT). Mobile edge computing and software-defined networks (SDN) are among the technologies that can be deployed in cellular networks for improving latency and security in public safety services [22]. Furthermore, cellular networks offer novel capabilities for the management of various use cases with varying priorities, such as network function virtualization and network slicing [5]. For instance, in the case of big events or major accidents, the traffic prioritization mechanisms allow the necessary network capacity and performance for first responders in the first place, and for other users in the second place [22].

Addressing new verticals and markets including the public safety industry, the 5G standard introduces several features that target different requirements and use cases. 5G networks can support a very large number of devices that can communicate with each other and exchange data. They are thus helping to address the IoT evolution. In 5G networks, IoT devices are expected to form significant sources of information for the public safety community [34]. By processing this information and integrating it into public safety operations, first responders can be more proactive and their tasks can be moved from investigation to prevention of accidents and crimes [5].

IoT device adoption among the public safety community will enable various use cases such as communication center alerting, accident video investigation, and connected and automated cars. Among these new examples, certain applications require very high data rates that can be achieved by the exploitation of techniques such as beamforming and small cells in 5G-enabled public safety networks [5]. Furthermore, 5G networks are expected to support network-based localization with an accuracy of less than 1 m [35]. This boost in localization accuracy, in comparison to LMR technologies, can help provide reliable and fast emergency responses through public safety networks [34]. Therefore, 5G-related 3GPP specifications are outlined to include various features and functionalities that can improve the services offered by next-generation public safety networks.

As discussed above, next-generation public safety networks can employ cellular solutions and technologies that are ratified by 3GPP in its technical specifications for both 4G and 5G standards. Hence, one can raise the question as to which network deployment option to select. Actually, several public safety agencies are already implementing LTE-based, next-generation public safety networks since not all mission-critical capabilities are fully supported by NR networks. It is possible for them to eventually upgrade their networks and support both 4G and 5G capabilities [36].

## 4. Wearable Technology in Next-Generation Public Safety Networks

With the evolution of public safety technologies and services, there is an increasing number of devices that are being deployed in these networks. After having only PTT devices in LMR networks, safety, security, and healthcare professionals are using cellular-enabled mission-critical smartphones, laptops, tablets, as well as connected vehicles in PS-LTE networks. With the aim of further extending public safety use cases, new devices are being deployed in a novel ecosystem of public safety communications, known as IoLST [37]. Similar to the general definition of IoT, the IoLST is a network of devices that collect data and use various communication technologies to share it in real-time. However, its purpose is specific and focuses on improving public safety responses to emergencies [37]. IoLST represents an extension of LMR and PS-LTE capabilities. In detail, IoLST solutions extend the public safety use cases with new types of applications including, but not limited to, real-time video sharing using body-worn cameras, traffic system control with sensor-equipped vehicles, healthcare and vital sign monitoring of first responders, and drone surveillance systems [38]. These IoLST use cases involve a variety of devices among which wearables are gaining the attention of the public safety community.

One characteristic of the public safety workforce is mobility. Public safety personnel across law enforcement, firefighting, and EMS primarily operate in the field and deal with dangerous situations outside of their response vehicles. Hence, relying on laptop computers is no longer an alternative for first responders to stay connected. Hands-free operation is another peculiarity of public safety services, where the personnel is generally equipped with protective gloves that make it difficult for them to hold smartphones or tablets. Regarding these challenges, the IoLST ecosystem involves wearable devices to make use of their form factor and their capability to encompass advanced sensors. Wearable-empowered IoLST aims to offer unique capabilities for the highly mobile workforce and mark an important shift in the daily operation of the public safety personnel [37].

**Figure 3:**
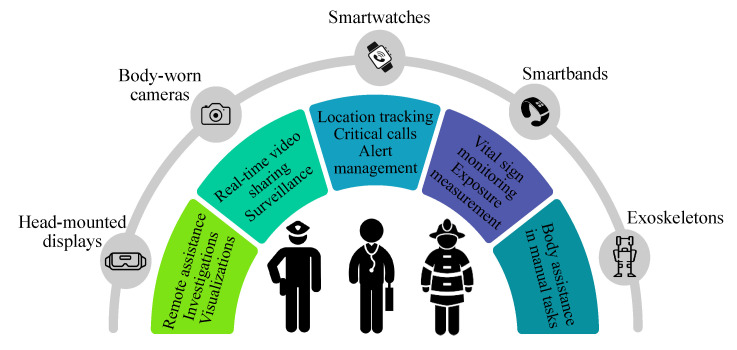
Examples of wearable-based public safety services.

Admittedly, wearables can complement other cellular-enabled devices and ensure that users stay closely connected to the data they need. Therefore, wearable technology can deliver reliable in-field communications, enhanced situational awareness, and improved first-responder safety. In Figure 3, we illustrate examples of IoLST use cases, where emergency first responders employ a multitude of wearable devices for various public safety applications. For instance, EMS workers in outdoor locations can use smart glasses for remote assistance from indoor experts and professional doctors. Furthermore, outfitting first responders with smart-bands enables the measurement of exposure to toxic substances and the monitoring of their vital signs (i.e., glucose, blood pressure, and heart rate) while operating in dangerous environments [39]. Other examples of wearable solutions for public safety can include smart gloves and exoskeletons for supporting manual tasks.

As depicted in Figure 3, several wearable devices can be utilized for the transmission of potentially life-saving communications in a large-scale emergency response. However, watch-type wearables are expected to be a game changer for public safety operations [40]. The ability to send real-time location, monitor alerts, make calls, and acknowledge the reception of messages while working allows first responders to perform their critical tasks in a safer and more responsive manner [40]. In terms of communication capabilities, several cellular-enabled smartwatches are currently available in the wearable market. Specifically, these devices utilize cellular low-power wide-area (LPWA) technologies, namely, LTE machine-type communications (LTE-M) and narrowband IoT (NB-IoT). These connectivity solutions can provide public safety applications at better coverage conditions, lower cost, and lower power consumption than conventional cellular technologies [37]. The deployment of cellular LPWA-enabled smartwatches will allow the public safety community to assess the opportunities provided by cellular connectivity and wearable technology.

## 5. Latency and Reliability Performance Evaluation in Wearable-Based MCPTT Applications

In this section, we investigate the latency and the reliability performance in wearable-based mission-critical applications, more precisely, off-network MCPTT services using cellular-enabled smartwatches. The deployed smartwatches belong to one of the LTE-M device categories (LTE Cat M1 and LTE Cat M2), specifically, LTE Cat M2. In the following subsections, we refer to an LTE Cat M2 smartwatch as a UE.

### 5.1. Evaluation Methodology and Parameters

We utilized network simulator 3 (ns-3) for the performance evaluation of MCPTT application requirements. Specifically, this evaluation was conducted based on the LTE-EPC network simulator (LENA) updated with D2D communications and MCPTT services. Such updates are supported by the research community and were first published in [41]. Further, we extend the LENA module with the features needed for the simulation of public safety scenarios and wearable devices. In detail, we consider scenarios in which the cellular network is deployed in the 700 MHz frequency band. In terms of wearable device modeling, an empirical off-body propagation loss model is implemented to better capture the signal propagation between wearable devices [42].

We also updated LENA’s adaptive modulation and coding model with the reduced baseband capabilities that are provided by the 3GPP physical layer specifications [43]. In 3GPP specifications, the term bandwidth-reduced low-complexity (BL) is used to indicate the implementation of LTE-M device categories. More precisely, 3GPP TS 36.213 provides the recommendations for complexity reduction and baseband configuration of these devices. As part of these recommendations, BL UEs have a maximum bandwidth of 5 MHz, a 16QAM maximum modulation scheme in both uplink (UL) and downlink (DL), and one Tx/Rx antenna. These and other wearable device-related parameters are declared in Table 3 along with D2D and MCPTT application-related parameters.

In terms of the network layout and the MCPTT functional mode adopted in this performance evaluation, we focus on off-network MCPTT services with out-of-coverage UEs. An illustration of the evaluation scenario can also be seen in Figure 1. As discussed in Section 3.1, out-of-coverage UEs are restricted to using sidelink transmission mode-2. Notably, the resource allocation approach without the network side’s assistance is widely applied in public safety scenarios [44]. Therefore, we utilize this autonomous mode for resource allocation in D2D communications as depicted in Table 3. MCPTT off-network mode supports several types of calls [45]. In this paper, we focus on MCPTT private and basic group calls. A private call is established between two MCPTT applications for two users to communicate. A basic group call is a call where a group of users that are associated with a particular group ID contend to talk. The term “basic” is used in 3GPP specifications to point out the difference between these types of calls and broadcast group calls. In the rest of the paper, MCPTT basic group calls will be simply referred to as MCPTT group calls. Five UEs participated in our MCPTT group calls.

Before a D2D communication takes place on the Physical Sidelink Shared Channel (PSSCH), a sidelink grant needs to be preconfigured. In sidelink transmission mode-2, the MCPTT call members are responsible for determining the modulation and coding scheme (MCS), the allocation size (i.e., number of resource blocks (RBs)), and the number of transmission opportunities in each time resource pattern defined by the kTRP parameter. These sidelink grant parameters are preconfigured in the devices, as stated in Table 3. The Physical Sidelink Control Channel (PSCCH) period parameter defines the periodicity of this grant configuration performed by each UE. It is also called sidelink period and can have one of these values: 40, 60, 80, 120, 140, 160, 240, 280, or 320 ms.

**Figure 4:**
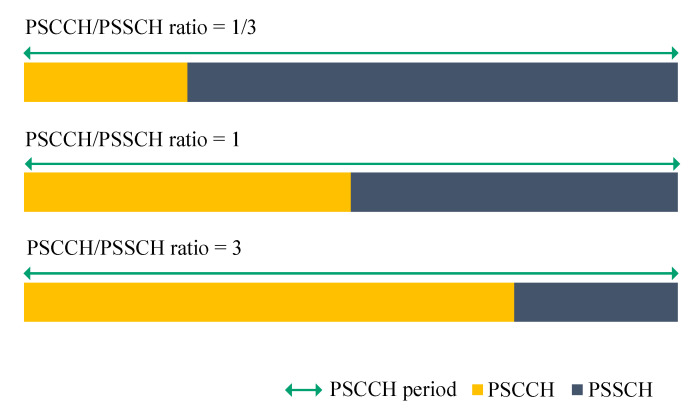
Sidelink channel configurations utilized in the performance evaluation.

Within a PSCCH period, there are separate subframes for control (PSCCH) and for data (PSSCH). Hence, the PSCCH to PSSCH (PSCCH/PSSCH) ratio needs to be fixed. This ratio reflects the distribution of sidelink resources in the time domain (i.e., number of subframes). An illustration of the three adopted sidelink channel configurations is provided in Figure 4. For instance, the same number of subframes is allocated for both sidelink channels in the second configuration (PSCCH/PSSCH ratio = 1). The two other configurations are based on the preference (i.e., allocation of more subframes) for one channel over the other. At the application level, the MCPTT model that we utilize in the evaluation assumes that 60-byte voice packets are generated every 20 ms. This means that the data rate demand for the voice communication is 24 kbps.

### 5.2. Performance Evaluation Results

After setting the scenarios and the parameters, the next step is to run several simulations and collect the simulation traces that will be used in the performance evaluation. As mentioned in Section 5.1, we use ns-3 updated with models for the support of public safety communications and presented in [41]. These models allow tracing MCPTT messages at the application layer and provide output files with these traces. The obtained output files contain the elements that we will use to determine our evaluation metrics.

**Figure 5:**
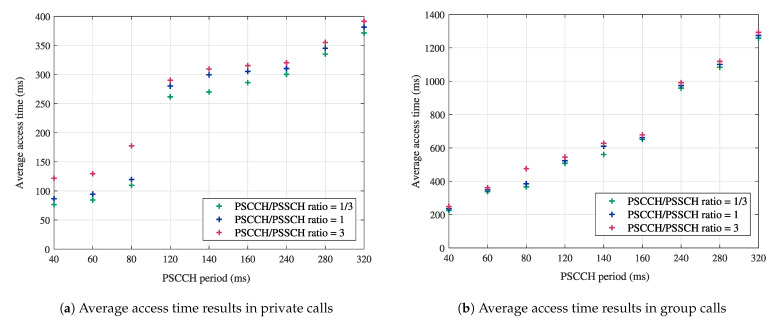
Impact of the PSCCH period and the PSCCH/PSSCH ratio on latency performance in MCPTT calls.

In the latency performance evaluation, we assess the MCPTT access time. According to 3GPP specifications, the MCPTT access time is defined as “the time between when an MCPTT user requests to speak and when this user gets a signal to start speaking” [45]. To determine this metric in group calls, we use the same calculation method detailed in [6]. For the MCPTT private calls, the access time was determined based on the formulas provided in [46]. Figure 5 reports the numerical results for the average access time in MCPTT private (Figure 5a) and group calls (Figure 5b). The provided results are in function of the PSCCH period and the PSCCH/PSSCH ratio. The values of both parameters are fixed as stated in Section 5.1.

The average access time results in private and group calls have different ranges due to the different number of UEs and the different call configurations (e.g., types of messages, counters, and timers). One common observation in Figure 5a,b is that longer PSCCH periods result in longer access time values. This can be justified by the fact that one important component of the access time is the “floor request retransmission” timer, which is equal to the PSCCH period according to the 3GPP default setting. The impact of the PSCCH/PSSCH ratio on the MCPTT access time is also depicted in Figure 5a,b. Although this impact is slightly clearer in Figure 5a, both figures show that preferring PSSCH over PSCCH transmissions (i.e., shorter PSCCH/PSSCH ratio) can provide shorter access time values. The latter result is important in public safety services where even a few milliseconds can make a difference in critical situations. In brief, we can summarize the take-away message from the obtained results in Figure 5 as follows: to guarantee short access times for MCPTT communications, short PSCCH periods with low PSCCH/PSSCH ratios are recommended.

**Figure 6:**
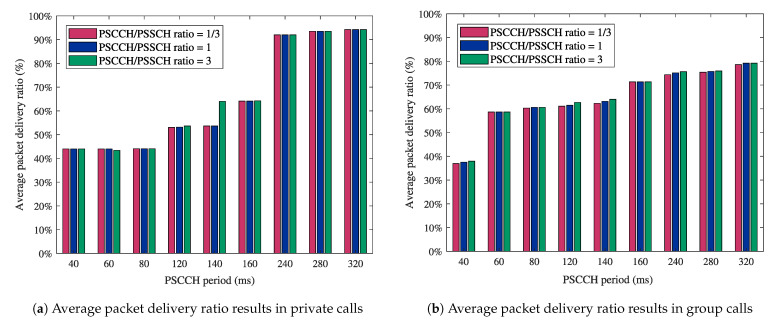
Impact of the PSCCH period and the PSCCH/PSSCH ratio on reliability performance in MCPTT calls.

The second part of this evaluation is dedicated to reliability performance. The packet delivery ratio is utilized as the reliability performance indicator. It can be defined as the ratio of the number of packets received at the destination to the number of packets sent from the source [47]. Figure 6 reports the numerical results for the average packet delivery ratio in MCPTT private (Figure 6a) and group calls (Figure 6b). Similar to Figure 5, the results provided in Figure 6 depend on the PSCCH period and the PSCCH/PSSCH ratio. Lower values of the packet delivery ratio are witnessed when using shorter PSCCH periods in both private and group calls. Short PSCCH periods implicate more reconfiguration times, and thus, an increase in the probability of collisions due to the frequent exchange of scheduling messages.

**Figure 7:**
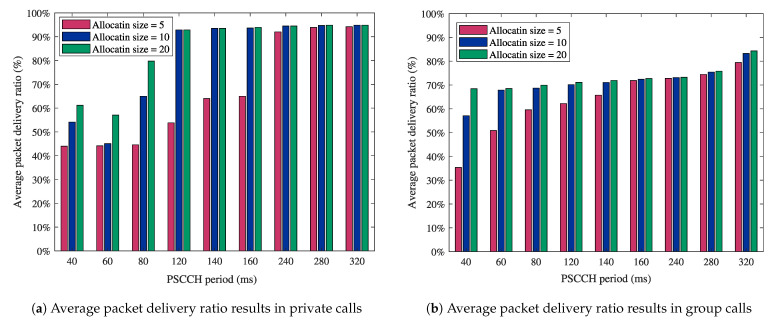
Impact of the sidelink allocation size on reliability performance in MCPTT calls.

On top of the PSCCH period and the number of PSCCH subframes, the collision probability of a PSCCH transmission depends on the sidelink allocation size [44]. Therefore, we examine the impact of the sidelink allocation size on the packet delivery ratio in MCPTT private (Figure 7a) and group calls (Figure 7b). The obtained results in Figure 7 show that high packet delivery ratios can be achieved using long PSCCH periods and a high number of RBs in the sidelink grant. However, this action has two limitations: (i) the increase in the MCPTT access time values due to the long PSCCH periods (as seen in Figure 5) and (ii) the allocation size constraint due to the reduced bandwidth of wearable devices. Hence, reliable MCPTT communications with high packet delivery ratios can be provided at the expense of the access time performance.

As a result of this performance evaluation part, we can deduce the need for latency and reliability trade-off mechanisms in wearable-based MCPTT applications. These findings can be useful for the standardization community to take into account the MCPTT reliability requirements. This is necessary, especially since current 3GPP specifications only consider latency-related metrics for the evaluation of MCPTT services. Additional incentives from the research community can investigate these trade-off mechanisms in MCX services, and thus, help further enable the support of reliable mission-critical applications over cellular systems.

### 5.3. Future Considerations

In this section, we discuss novel 3GPP standardization works that can be further considered in latency and reliability performance evaluations for wearable-based MCPTT applications. The first consideration is related to the baseband and radio-frequency capabilities of cellular-enabled wearable devices. As a matter of fact, LPWA-enabled wearables are not the only standalone wearable devices that can utilize cellular connectivity. Wearables are among the targeted use cases in beyond-5G systems. This can be confirmed by the introduction of the NR reduced-capability (NR RedCap) technology in 3GPP Rel-17 [48]. Table 4 summarizes the main capabilities of an LTE Cat M2 device compared to those of a RedCap device operating in the 5G frequency range 1 (FR1). With reference to our performance evaluation, NR RedCap-enabled smartwatches can have higher bandwidth, which implicates the possibility of configuring sidelink grants with a higher number of RBs. This will affect reliability performance, as discussed in Section 5.2.

The impacts of these reduced device capabilities on the network performance and needed recovery strategies are studied in the 3GPP Rel-17 specifications on NR RedCap [49]. The latest recommendations can help to gain new insights into the performance evaluation of several requirements in wearable-based, mission-critical applications. Another fact that can motivate public safety agencies to integrate NR RedCap-enabled wearables in their networks is that, with cloud native technologies being central parts of the 5G core architecture, the network will provide wearable devices with the needed storage capacity and processing power [50]. Hence, NR RedCap-enabled wearables will be able to host more sensors, collect more data, and be involved in new sets of public safety applications.

Other essential considerations are related to NR sidelink enhancements for public safety that are planned for 3GPP Rel-17. As discussed in Section 3.1, although the first standard for NR sidelink in Rel-16 provides solutions specified mainly for V2X services, some of these solutions can also be utilized for public safety applications with additional enhancements [51]. Among these, we focus on the resource allocation enhancements in sidelink transmission mode-2. The latest 3GPP work items on NR sidelink enhancements propose the definition of new resource selection or grant scheduling methods [51]. In the above performance evaluation, we use a fixed grant scheduling method where the sidelink grant parameters (i.e., MCS, allocation size, and kTRP) are preconfigured in the simulations. Nevertheless, the determination of these parameters by out-of-coverage UEs can be performed randomly (i.e., random scheduling) or following certain optimization goals. The latter include selecting a grant configuration that utilizes the minimum number of RBs per transmission (i.e., min. RB scheduling) or maximizes the communication range (i.e., max. coverage scheduling).

**Figure 8:**
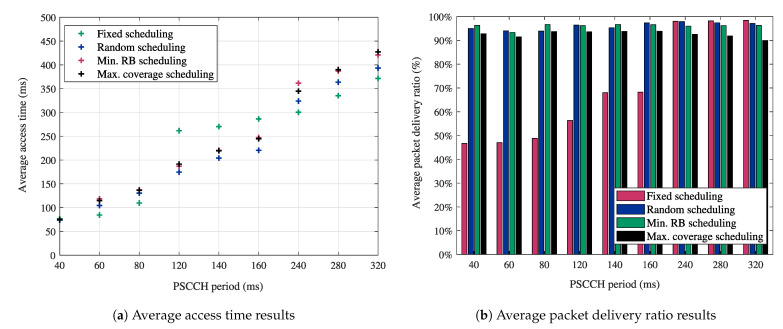
Impact of the resource selection methods in sidelink transmission mode-2 on latency and reliability performance in MCPTT private calls.

To better understand the significance of these sidelink grant scheduling methods in MCPTT applications, we present preliminary results based on our simulations described in Section 5.1. Figure 8 reports the numerical results for the average access time (Figure 8a) and the average packet delivery ratio (Figure 8b) in the function of the PSCCH period and the resource selection method in MCPTT private calls. Figure 8a,b show that for each value of the PSCCH period, there is a scheduling method that provides better results than the other methods in terms of latency and reliability, respectively. The impact of resource selection methods is even clearer when considering MCPTT group calls with an increased number of users [6]. The obtained early-stage evaluation results show potential insights that motivate future standalone studies on the choice of suitable resource selection methods in sidelink transmission mode-2.

The latest 3GPP work items on NR sidelink enhancements recommend also the study of the feasibility and benefits of new solutions for improved reliability and reduced latency in sidelink transmission mode-2. The main solution that is defined in these items is inter-UE coordination [52]. In brief, a UE A determines a set of resources for the sidelink transmission and sends it to a UE B in mode-2, which takes the received set into account in the resource selection for its own transmission. The sent set of resources from UE A to UE B can be based on sensing results and/or potential resource conflicts. The final solution should be able to operate in all coverage scenarios and further studies are required to assess its feasibility and benefits in terms of latency and reliability [51].

## 6. Conclusions

The migration from LMR systems to next-generation public safety networks represents a significant opportunity for public safety agencies to enhance office-bound applications and enable new mission-critical solutions. By adopting standardized communication technologies, public safety organizations can have access not only to a portfolio of cellular features and capabilities but also to continuous innovations and enhancements. To better understand the opportunities brought by 4G and 5G technologies for public safety operations, we provided a state-of-the-art overview of cellular solutions ratified by 3GPP to enable the deployment of next-generation public safety networks. In addition to communication technologies, we addressed another key factor in public safety operations, which are devices and assistant systems used by first responders. Specifically, we focused on wearable devices as part of a cellular-enabled IoLST ecosystem. We provided examples of use cases that show how wearable technology can help in delivering improved safety and situational awareness for first responders.

Motivated by the potential role of watch-type wearables in future public safety operations, we evaluated latency and reliability performance in MCPTT applications using LTE Cat M2 smartwatches. More precisely, we examined access time and packet delivery ratio with different combinations of sidelink-related parameters in MCPTT private and group calls. The goal of this evaluation was to shed light on the need for latency and reliability trade-off mechanisms in mission-critical applications. Additionally, we discussed novel 3GPP standardization efforts that can be explored in an extended version of the former performance evaluation. The identified future considerations can allow for a deeper understanding of the support of wearable-based, mission-critical application requirements over next-generation public safety networks.

## Figures and Tables

**Table 1:** Main cellular solutions for public safety.

Technology	3GPP Release	Brief Description
GCSE	Rel-12	Mechanisms enabling both unicast and multicast transmissions for group communications
ProSe	Rel-12	Architecture and radio interface for the support of D2D communications
IOPS	Rel-13	Isolated mode of operation that ensures communications between users via base stations without backhaul links
MCPTT	Rel-13	Delivering voice services for public safety users
MCData	Rel-14	Delivering non-real-time data services for public safety users
MCVideo	Rel-14	Delivering real-time video services for public safety users

**Table 2:** Enhancements of public-safety-targeted cellular solutions.

Technology	3GPP Releases	Main Improvements
ProSe	Rel-13–Rel-16	ProSe discovery and direct communication features in EPS, UE-to-network relaying for IoT and wearables
	Rel-17	Support of NR ProSe communications and UE-to-network relay over 5G networks
MCX	Rel-14	User authentication, service authorization, media and control signaling encryption
	Rel-15	Support of MBMS, interworking with LMR systems, and interconnection between cellular mission-critical systems
	Rel-16–Rel-17	Enhanced procedures and information flows in on-network and off-network architectures, identification of further requirements of MCX services

**Table 3:** Performance evaluation parameters.

**Wearable Device-Related Parameters**	**Value**
UE category	LTE Cat M2
Max. bandwidth	5 MHz
Max. UL/DL modulation order	4 (16-QAM)
UE transmission mode	1 (1 Tx/Rx antenna)
UE power class	5
UE transmit power	20 dBm
UE noise figure	9 dB
**D2D-Related Parameters**	**Value**
Sidelink transmission mode	Mode-2 (autonomous)
Sidelink MCS	10
Sidelink allocation size	5 RBs
kTRP	1
PSCCH period	40 ms
PSCCH/PSSCH ratio	1/3, 1, 3
**MCPTT Application-Related Parameters**	**Value**
Functional mode	Off-network
Types of calls	private and basic group calls
Message size	60 bytes
Inter-packet interval	20 ms

**Table 4:** Main capabilities of LTE Cat M2 and NR RedCap devices.

Device Capability	LTE Cat M2	NR RedCap
Max. bandwidth	5 MHz	20 MHz
Max. UL modulation order	4 (16QAM)	4 (16QAM)
Max. DL modulation order	4 (16QAM)	6 (64QAM)
UE transmission mode	1 (1 Tx/Rx antenna)	1 (1 Tx/Rx antenna)
UE power class	5	3
UE transmit power	20 dBm	23 dBm

## Data Availability

Not applicable.

## References

[1] Yu W., Xu H., Nguyen J., Blasch E., Hematian A., Gao W. (2018). Survey of public safety communications: User-side and network-side solutions and future directions. IEEE Access.

[2] Chaudhry A.U., Hafez R.H. (2019). LMR and LTE for public safety in 700 MHz spectrum. Wirel. Commun. Mob. Comput..

[3] Stojkovic M. (2016). Public Safety Networks towards Mission Critical Mobile Broadband Networks. Master’s Thesis.

[4] (2020). Enabling Intelligent Operations with Mission Critical Networks.

[5] 4G and 5G for Public Safety. https://tcca.info/documents/2017-march_tcca_4g_and_5g_for_-public_safety.pdf/.

[6] Saafi S., Hosek J., Kolackova A. Cellular-enabled Wearables in Public Safety Networks: State of the Art and Performance Evaluation. Proceedings of the 2020 12th International Congress on Ultra Modern Telecommunications and Control Systems and Workshops (ICUMT).

[7] Baldini G., Karanasios S., Allen D., Vergari F. (2013). Survey of wireless communication technologies for public safety. IEEE Commun. Surv. Tutor..

[8] Yarali A. (2020). Public Safety Networks from TETRA to Commercial Cellular Networks. Public Safety Networks from LTE to 5G.

[9] Kumbhar A., Koohifar F., Güvenç I., Mueller B. (2016). A survey on legacy and emerging technologies for public safety communications. IEEE Commun. Surv. Tutor..

[10] Doumi T., Dolan M.F., Tatesh S., Casati A., Tsirtsis G., Anchan K., Flore D. (2013). LTE for public safety networks. IEEE Commun. Mag..

[11] Jarwan A., Sabbah A., Ibnkahla M., Issa O. (2019). LTE-based public safety networks: A survey. IEEE Commun. Surv. Tutor..

[12] Yarali A. (2020). 4G and 5G for PS. Public Safety Networks from LTE to 5G.

[13] Lessi C.C., Chochliouros I.P., Trakadas P., Karkazis P. (2021). Advanced First Responders’ Services by Using FASTER Project Architectural Solution. Proceedings of the IFIP International Conference on Artificial Intelligence Applications and Innovations.

[14] First Responder Advanced Technologies for Safe and efficienT Emergency Response. https://www.faster-project.eu/.

[15] Spada M.R., Pérez-Romero J., Sanchoyerto A., Solozabal R., Kourtis M.A., Riccobene V. Management of mission critical public safety applications: The 5G ESSENCE project. Proceedings of the 2019 European Conference on Networks and Communications (EuCNC).

[16] 5G ESSENCE|Embedded Network Services for 5G Experiences. https://www.5g-essence-h2020.eu/.

[17] Merwaday A., Guvenc I. UAV assisted heterogeneous networks for public safety communications. Proceedings of the 2015 IEEE Wireless Communications and Networking Conference Workshops (WCNCW).

[18] Naqvi S.A.R., Hassan S.A., Pervaiz H., Ni Q. (2018). Drone-aided communication as a key enabler for 5G and resilient public safety networks. IEEE Commun. Mag..

[19] Seo S., Kim S., Kim S.L. A public safety framework for immersive aerial monitoring through 5G commercial network. Proceedings of the 2020 IEEE Wireless Communications and Networking Conference Workshops (WCNCW).

[20] Alsamhi S.H., Ma O., Ansari M.S., Gupta S.K. (2019). Collaboration of Drone and Internet of Public Safety Things in Smart Cities: An Overview of QoS and Network Performance Optimization. Drones.

[21] Fodor G., Dahlman E., Mildh G., Parkvall S., Reider N., Miklós G., Turányi Z. (2012). Design aspects of network assisted device-to-device communications. IEEE Commun. Mag..

[22] Yarali A. (2020). Public Safety Communications Evolution. Public Safety Networks from LTE to 5G.

[23] (2014). Study on LTE Device to Device Proximity Services; Radio Aspects (Release 12).

[24] Oueis J., Conan V., Lavaux D., Stanica R., Valois F. (2017). Overview of LTE isolated E-UTRAN operation for public safety. IEEE Commun. Stand. Mag..

[25] Mission-Critical Services in 3GPP. https://www.3gpp.org/news-events/3gpp-news/1875-mc_services.

[26] (2016). Common Functional Architecture to Support Mission Critical Services; Stage 2 (Release 14).

[27] (2020). Proximity-Based Services (ProSe); Stage 2 (Release 16).

[28] (2017). Study on Further Enhancements to LTE Device to Device (D2D), User Equipment (UE) to Network Relays for Internet of Things (IoT) and Wearables (Release 14).

[29] (2019). Study on System Enhancement for Proximity Based Services (ProSe) in the 5G System (5GS) (Release 17).

[30] (2016). Security of the Mission Critical Service (MCX) (Release 14).

[31] Guttman E. 3GPP Advances in Critical Communications. Proceedings of the Critical Communications World 2018 (CCW 2018).

[32] (2019). Common Functional Architecture to Support Mission Critical Services; Stage 2 (Release 17).

[33] (2019). Mission Critical Services Common Requirements (MCCoRe); Stage 1 (Release 17).

[34] Yarali A. (2020). Higher Generation of Mobile Communications and Public Safety. Public Safety Networks from LTE to 5G.

[35] Zhang P., Lu J., Wang Y., Wang Q. (2017). Cooperative localization in 5G networks: A survey. ICT Express.

[36] Bhatia B. Status and Trends of Public Protection and Disaster Relief (PPDR) Communications. https://www.itu.int/dms_pub/itu-r/oth/0a/0E/R0A0E0000CB0001PDFE.pdf.

[37] What Is the Internet of Life Saving Things (IoLST)?. https://www.sierrawireless.com/iot-blog/iot-blog/2018/12/internet-of-life-saving-things/.

[38] The Internet of Lifesaving Things: Smarter Cities, Smarter Response. https://about.att.com/newsroom/internet_of_lifesaving_things.html.

[39] (2019). Enhancing Response Capabilities with Smartwatches in Public Safety.

[40] CAD on Smartwatches Is a Game Changer for Police Communications. https://www.publicsafety.network/blog-5.29.19.html.

[41] Rouil R., Cintrón F.J., Ben Mosbah A., Gamboa S. Implementation and Validation of an LTE D2D Model for ns-3. Proceedings of the Workshop on ns-3.

[42] Garcia-Serna R.G., Garcia-Pardo C., Molina-Garcia-Pardo J.M. (2015). Effect of the receiver attachment position on ultrawideband off-body channels. IEEE Antennas Wirel. Propag. Lett..

[43] (2007). Evolved Universal Terrestrial Radio Access (E-UTRA); Physical Layer Procedures (Release 8).

[44] Shih M.J., Liu H.H., Shen W.D., Wei H.Y. UE autonomous resource selection for D2D communications: Explicit vs. implicit approaches. Proceedings of the 2016 IEEE Conference on Standards for Communications and Networking (CSCN).

[45] (2014). Mission Critical Push to Talk (MCPTT); Stage1 (Release 13).

[46] Sun Y., Garey W., Rouil R., Varin P. (2019). Access time analysis of MCPTT off-network mode over LTE. Wirel. Commun. Mob. Comput..

[47] Khairnar M.V.D., Kotecha K. (2013). Simulation-Based Performance Evaluation of Routing Protocols in Vehicular Ad-hoc Network. Int. J. Sci. Res. Publ..

[48] (2020). Study on Support of Reduced Capability NR Devices (Release 17).

[49] (2020). Potential UE complexity reduction features for RedCap.

[50] (2019). 5G and The Cloud.

[51] (2020). NR Sidelink Enhancement.

[52] (2020). LS on Mode 2 Enhancements in NR Sidelink.

